# Early Development of the Central and Peripheral Nervous Systems Is Coordinated by Wnt and BMP Signals

**DOI:** 10.1371/journal.pone.0001625

**Published:** 2008-02-20

**Authors:** Cédric Patthey, Lena Gunhaga, Thomas Edlund

**Affiliations:** Umeå Center for Molecular Medicine, Umeå University, Umeå, Sweden; Medical College of Georgia, United States of America

## Abstract

The formation of functional neural circuits that process sensory information requires coordinated development of the central and peripheral nervous systems derived from neural plate and neural plate border cells, respectively. Neural plate, neural crest and rostral placodal cells are all specified at the late gastrula stage. How the early development of the central and peripheral nervous systems are coordinated remains, however, poorly understood. Previous results have provided evidence that at the late gastrula stage, graded Wnt signals impose rostrocaudal character on neural plate cells, and Bone Morphogenetic Protein (BMP) signals specify olfactory and lens placodal cells at rostral forebrain levels. By using in vitro assays of neural crest and placodal cell differentiation, we now provide evidence that Wnt signals impose caudal character on neural plate border cells at the late gastrula stage, and that under these conditions, BMP signals induce neural crest instead of rostral placodal cells. We also provide evidence that both caudal neural and caudal neural plate border cells become independent of further exposure to Wnt signals at the head fold stage. Thus, the status of Wnt signaling in ectodermal cells at the late gastrula stage regulates the rostrocaudal patterning of both neural plate and neural plate border, providing a coordinated spatial and temporal control of the early development of the central and peripheral nervous systems.

## Introduction

Information from our surroundings is transmitted by specific sensory neurons in the peripheral nervous system to the central nervous system were it is processed. Early in development secreted signals specify various cell types of both the central and peripheral nervous systems, which later will establish complex neural circuits that process sensory information. The mechanism by which the early development of the two nervous systems is temporally and spatially coordinated is poorly understood.

The peripheral nervous system arises from neural crest and placodal cells derived from neural plate border cells. Neural crest cells, which contribute to a vast array of cell types are generated along the entire rostrocaudal neuraxis except at rostral forebrain levels, where neural plate border cells generate placodal but no neural crest cells [1]–[3]. In chick, the specification of both neural crest and placodal cells is ongoing at the late gastrula stage [4], [5], and around this stage, *Bmp2* and *Bmp4* are expressed in the ectoderm surrounding the entire neural plate [6], domains where phosphorylated Smad-1 is also detected, indicative of active BMP signaling [7]. At the late gastrula stage, BMP signals induce olfactory and lens placodal character in neural plate border cells at rostral forebrain levels [5]. Studies conducted later in development, at neural fold stages, when border cells have started to express neural crest markers [8], [9] suggest that both BMP and Wnt signals induce neural crest character in caudal neural cells [10]–[12]. Since neural crest generation has already been initiated at neural fold stages, these results may reflect mechanisms of regeneration and maintenance of inducing capacity rather than the mechanism by which neural crest cells are initially induced. Thus, it remains unclear whether BMP and Wnt signals act in parallel or have separated roles during the initial induction of neural crest cells.

Members of the *Wnt* family as well as members of the Wnt receptor family Frizzled are expressed in the caudal region of the gastrula stage embryo, and Wnt inhibitors are expressed in more rostral regions of the embryo [13]. In agreement with this spatial pattern of expression it has been shown that Wnt signals act in a graded manner to specify neural plate cells of progressively more caudal character [14], [15]. The rostrocaudal distribution of Wnts and Wnt inhibitors at the late gastrula stage raises the possibility that Wnt signals also impose a caudal character on neural plate border cells which may influence the specification of placodal and neural crest cells.

To address how Wnt and BMP signaling interact during the initial specification of olfactory/lens placodal and neural crest cells, we established explant assays of neural crest and placodal cell differentiation using late gastrula stage chick embryos. We now provide evidence that at the late gastrula stage, Wnt signals impose caudal character on neural plate border cells and that under these conditions, BMP signals induce neural crest cells instead of rostral placodal cells. Thus, the status of Wnt signaling in ectodermal cells at the late gastrula stage regulates the rostrocaudal patterning of both neural and neural plate border cells, providing a coordinated spatial and temporal control of the early development of the central and peripheral nervous systems.

## Results

### BMP Activity is Required for the Specification of Neural Crest Cells at the Late Gastrula Stage

**Figure 1:**
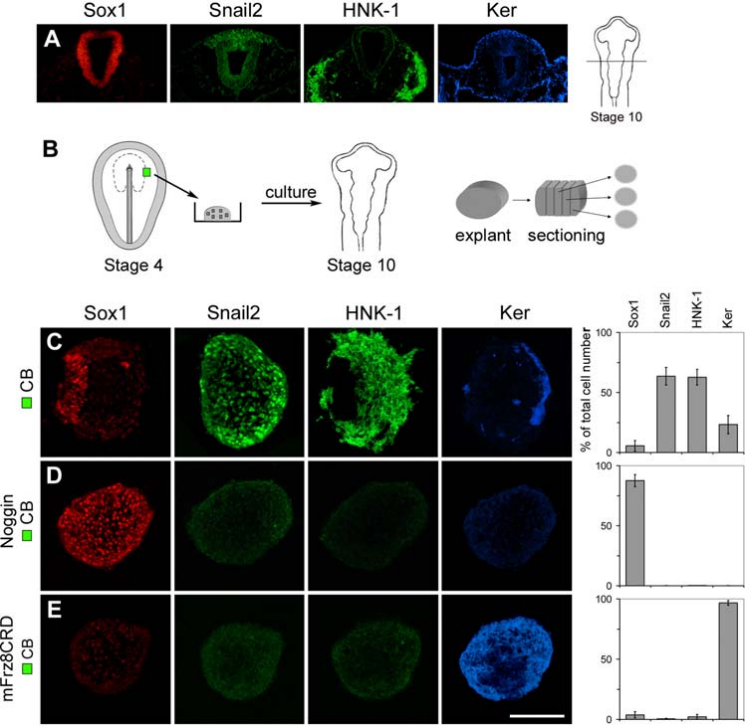
BMP and Wnt Signals are Required for the Specification of Neural Crest at the Gastrula Stage. (A) To the right, a schematic stage 10 chick embryo. The line indicates the level of the transverse sections shown in the corresponding panel. Sox1 is expressed in neural cells. Snail2 is expressed in premigratory and early migratory neural crest cells. HNK-1 is expressed in all migratory neural crest cells. Ker is expressed in epidermal cells. (B) Ectodermal explants were isolated, separated form the mesoderm and endoderm, cultured in vitro to the developmental equivalent of stage 10, before fixation, freezing and sectioning. Green box indicates explanted tissue used in (C–E). (C–E) Consecutive sections showing expression of molecular markers in explants cultured for 20–22 hr. (C) Stage 4 CB explants (n = 30) generated Snail2^+^ and HNK-1^+^ cells, and a few Sox1^+^ cells and Ker^+^ cells. (D) Stage 4 CB explants cultured in the presence of Noggin (n = 30) generated Sox1^+^ cells, but no Snail2^+^, HNK-1^+^ or Ker^+^ cells. (E) Stage 4 CB explants cultured in the presence of mFrz8CRD (n = 20) generated Ker^+^ cells, but no Sox1^+^, Snail2^+^ or HNK-1^+^ cells. Data are represented as mean±SEM. Scale bar, 100 µm (C–E).

In chick embryos, the specification of neural crest cells has been initiated at the late gastrula stage, stage 4 [4]. By stage 10, Snail2 (previously known as Slug) is preferentially expressed in pre-migratory and early migratory neural crest cells, and HNK-1 is expressed in all migratory neural crest [16] (Fig. 1A). Sox1 is specifically expressed in neural cells (Fig. 1A), and cells in the midbrain-hindbrain region express in addition Pax2 and/or En1/2 [17], [18]. Cytokeratins (Ker) are expressed in epidermal ectoderm (Fig. 1A) and in cranial placodes [19]. By stage 17 (E2.5) cells in the olfactory placode express Ker and *Raldh3* and a subset of cells express HuCD, while cells in the lens express Ker and δ-crystallin [5].

Recent results have provided evidence that the specification of neural crest cells has been initiated at stage 4 [4]. To elucidate the mechanism by which border cells become specified as neural crest cells, we established an explant assay of neural crest cell differentiation by culturing ectodermal explants of the neural plate border region of stage 4 chick embryos for 20–22 hr, corresponding in time to approximately stage 10 (Fig. 1B). The underlying mesoderm and endoderm were removed to avoid indirect effects from these germ layers. Explants of the caudal border (CB) region isolated at the prospective midbrain-hindbrain level of stage 4 embryos generated Snail2^+^ and HNK-1^+^ neural crest cells, but no or only a few Sox1^+^ neural and Ker^+^ epidermal cells (Fig. 1C). No mesodermal cells, herein analyzed by the expression of *Chordin*, *Brachyury*, *Tbx6L* and *Raldh2*, were detected (Fig. S1A and S1E). After prolonged culture (30 hr), corresponding in time to approximately stage 12 (E2), migratory cells characteristic of neural crest cells were generated (Fig. S2A). Thus, cells in the caudal border region are specified as neural crest cells at stage 4.

Both BMP and Wnt signals have been implicated in the generation of neural crest cells at the neural fold stage when neural plate border cells have started to express neural crest markers [20], [21]. We examined therefore first whether BMP signals are required for the initial induction of neural crest cells at the late gastrula stage, by culturing stage 4 CB explants in the presence of a selective antagonist of BMP signals. Under these conditions, the BMP inhibitor Noggin [22], blocked the generation of Snail2^+^ and HNK-1^+^ cells, and most cells acquired neural midbrain-hindbrain character (Fig. 1D, S2B and S2C). No significant differences in cell proliferation or apoptosis were detected in explants cultured alone compared to explants exposed to Noggin (Fig. S3A). Thus, at stage 4, BMP signals are required for prospective caudal border cells to acquire neural crest character, and in the absence of BMP activity cells acquire a caudal neural character.

### Neural Crest Cells Acquire Olfactory/Lens Placodal Fate in the Absence of Wnt Activity

**Figure 2:**
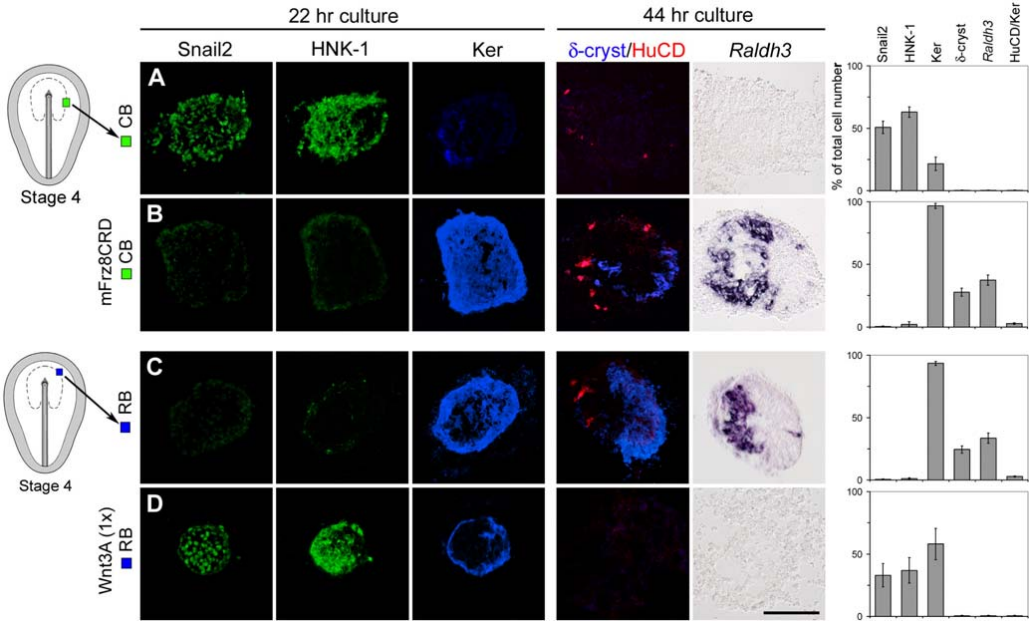
Wnt Activity Caudalizes Border Cells. (A–D) Consecutive sections showing expression of molecular markers in explants cultured for 20–22 hr or 43–45 hr. (A) Stage 4 CB explants (n = 30) generated Snail2^+^ and HNK-1^+^ cells, and a few Ker^+^ cells, but no δ-crystallin^+^, HuCD^+^ or *Raldh3* ^+^ placodal cells. (B) Stage 4 CB explants cultured in the presence of mFrz8CRD (n = 20) generated a distinct region of *Raldh3* ^+^, HuCD^+^ and Ker^+^ cells and a separate region of δ-crystallin^+^ and Ker^+^ cells, but no Snail2^+^ or HNK-1^+^ cells were detected. (C) Stage 4 RB explants (n = 30) generated a distinct region of *Raldh3* ^+^, HuCD^+^ and Ker^+^ cells and a separate region of δ-crystallin^+^ and Ker^+^ cells, but no Snail2^+^or HNK-1^+^cells were detected. (D) Stage 4 RB explants cultured in the presence of Wnt3A (1×) (n = 20) generated Snail2^+^ and HNK-1^+^cells, and a few Ker^+^cells, but no δ-crystallin^+^, HuCD^+^ or *Raldh3* ^+^ cells. Data are represented as mean±SEM. Scale bar, 100 µm (A–D).

To examine whether Wnt signals are required for the initial induction of neural crest cells at the late gastrula stage, we cultured stage 4 CB explants in the presence of soluble mFrz8CRD, which blocks Wnt, but not BMP signaling [23]–[25]. Exposure of mFrz8CRD also blocked the generation of Snail2^+^ and HNK-1^+^ cells, while Ker^+^ cells but no Sox1^+^ neural cells were generated (Fig. 1E). No significant differences in cell proliferation or apoptosis were detected in explants cultured alone compared to explants exposed to mFrz8CRD (Fig. S3B). To further define the identity of the Ker^+^ non-neural cells, stage 4 CB explants were cultured in the presence of mFrz8CRD for 43–45 hr, corresponding in time to approximately E2.5. Under these conditions, Ker^+^, *Raldh3* ^+^ and a few HuCD^+^ cells characteristic of the olfactory placode, and Ker^+^, δ-crystallin^+^ cells characteristic of the lens placode were generated in distinct non-overlapping regions of the explants (Fig. 2A and 2B). Thus at stage 4, when Wnt signaling is suppressed in prospective caudal neural plate border cells, the generation of neural crest cells is blocked and cells acquire olfactory and lens placodal character characteristic of cells derived from the rostral border.

### Wnt Signals Caudalize Neural Plate Border Cells

The finding that prospective neural crest cells acquire olfactory and lens placodal character in the absence of Wnt activity, indicates that Wnt signals impose caudal character on border cells. Previous results have provided evidence that at stage 4, cells of the rostral border located at the level of the prospective forebrain are specified as olfactory and lens placodal cells [5]. To test whether Wnt activity is sufficient to induce caudal border character in rostral border cells fated to generate lens and olfactory placodal cells, we exposed stage 4 prospective rostral border (RB) explants to Wnt3A (1×) for 20–22 hr and 43–45 hr. Wnt3A, Wnt8C and Wnt 11 show similar activities in several different assays (http://www.stanford.edu/7Ernusse/wntwindow.html). In the present study, the effects of Wnt signaling were examined by using Wnt3A conditioned medium, previously shown to have reliable biological activity [15], [23], [25]. In the presence of Wnt3A, Snail2^+^ and HNK-1^+^ neural crest cells were induced in stage 4 RB explants, while the generation of Ker^+^ cells was reduced and the generation of δ-crystallin^+^, *Raldh3* ^+^ and HuCD^+^ cells was blocked (Fig. 2C and 2D). No mesodermal cells were detected in explants exposed to Wnt3A (Fig. S1B) and no significant differences in cell proliferation or apoptosis were detected in explants exposed to Wnt3A compared to explants cultured alone (Fig. S3C). In summary, these results provide evidence that Wnt signals impose caudal character on neural plate border cells.

### BMP but not Wnt Signals Induce Neural Crest Character in Caudal Neural Cells

**Figure 3:**
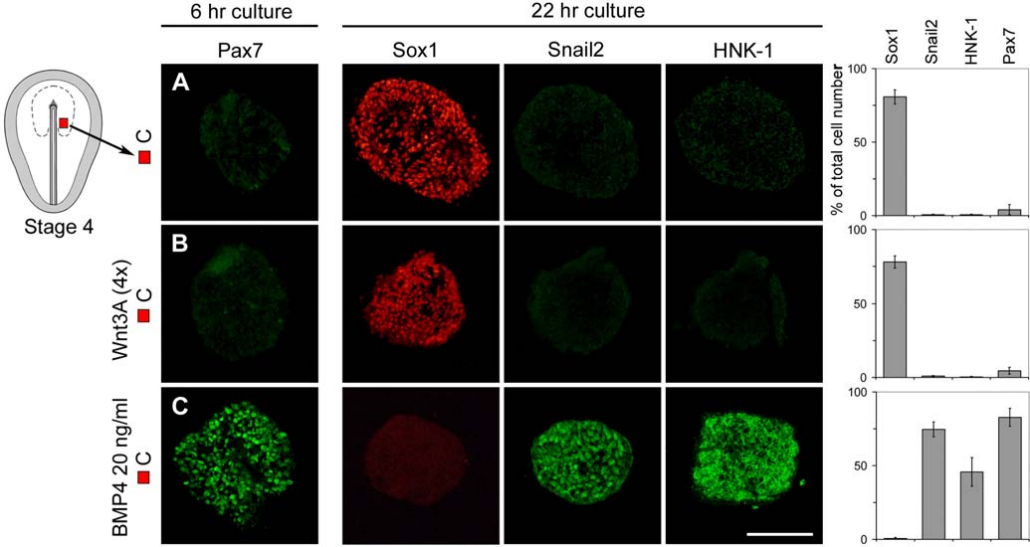
BMP but not Wnt Signals Induce Neural Crest Character in Caudal Neural Cells. (A–C) Consecutive sections showing expression of molecular markers in explants cultured for 6 hr or 20–22 hr. (A,B) Stage 4 C explants cultured alone (n = 25) or together with Wnt3A (4x) (n = 30) generated Sox1^+^cells, but no Snail2^+^, HNK-1^+^ or Pax7^+^ cells. (C) Stage 4 C explants cultured together with BMP4 (20 ng/ml) (n = 30) generated Pax7^+^, Snail2^+^and HNK-1^+^cells, but no Sox1^+^cells. Data are represented as mean±SEM. Scale bar, 100 µm (A–C).

We next tested whether Wnt and/or BMP signals are sufficient to induce neural crest fate in prospective caudal neural plate cells. To examine this issue we exposed stage 4 caudal (C) neural plate explants, fated to become midbrain-hindbrain cells, to Wnt3A or to BMP4. Exposure of stage 4 C explants to Wnt3A (1×-4×), increased nuclear staining of β-catenin, indicating activation of the canonical Wnt pathway (Fig. S4A and S4B; data not shown). However, Wnt3A did not block the generation of Sox1^+^ neural cells, and no Snail2^+^ or HNK-1^+^cells were induced (Fig. 3A and 3B). In contrast, exposure of stage 4 C explants to BMP4 (20 ng/ml) inhibited the generation of Sox1^+^ midbrain-hindbrain neural cells and induced a large number of Snail2^+^ and HNK-1^+^neural crest cells (Fig. 3C). No mesodermal cells were detected in explants exposed to BMP4 (Fig. S1C) and no significant differences in cell proliferation or apoptosis were detected in explants exposed to BMP4 compared to explants cultured alone (Fig. S3D). Thus, BMP but not Wnt signals induce neural crest fate in prospective caudal neural cells at the late gastrula stage. At head-fold stage, stage 6, prospective neural crest cells express Pax7 [4]. To provide further evidence that BMP, but not Wnt signals directly induce neural crest progenitors, we exposed stage 4 C explants to BMP4 (20 ng/ml) or Wnt3A for only 6h, corresponding in time to approximately stage 6. Under these conditions, BMP4 induced Pax7^+^ cells in stage 4 C explants, characteristic of neural crest progenitor cells (Fig. 3C) whereas explants cultured alone or in the presence of Wnt3A did not generate Pax7^+^ cells (Fig. 3A and 3B). Thus, at the late gastrula stage, BMP but not Wnt signals induce neural crest fate in prospective caudal neural cells.

### Neural Crest Progenitor Cells Become Independent of Further Exposure to BMP and Wnt Signals at Head Fold Stages

**Figure 4:**
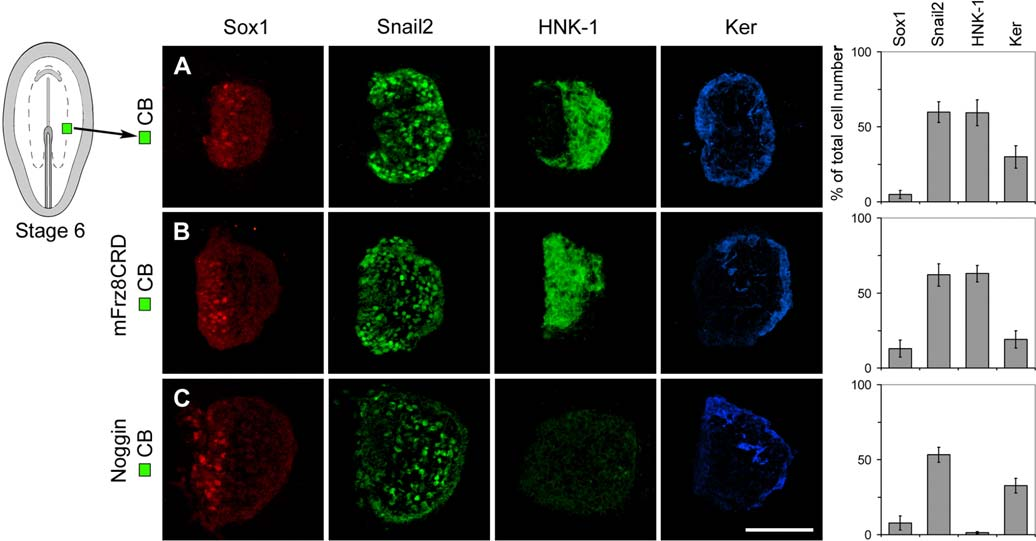
The Specification of Neural Crest is Independent of BMP and Wnt Activity at the Head Fold Stage. (A–C) Consecutive sections showing expression of molecular markers in explants cultured for 18–20 hr. (A,B) Stage 6 CB explants cultured alone (n = 20) or in the presence of mFrz8CRD (n = 15) generated Snail2^+^and HNK-1^+^cells, and a few Sox1^+^cells and Ker^+^cells. (C) Stage 6 CB explants cultured in the presence of Noggin (n = 20) generated Snail2^+^cells, and a few Sox1^+^cells and Ker^+^cells, but no HNK-1^+^ cells. Data are represented as mean±SEM. Scale bar, 100 µm (A–C).

At stage 4, Wnt signals impose caudal character on prospective neural plate cells [15] as well as on prospective border cells. By stage 6, the generation of caudal neural cells has become independent of exposure to Wnt signals [26]. We tested therefore whether by stage 6 the generation of neural crest cells also has become independent of Wnt activity, by exposing stage 6 CB explants isolated at the midbrain-hindbrain level to mFrz8CRD for 18–20 hr. Under these conditions, stage 6 CB explants still generated Snail2^+^ and HNK-1^+^ neural crest cells (Fig. 4A and 4B). In agreement with previous results performed at the trunk level [27], at this stage inhibition of BMP activity by Noggin inhibited the generation of HNK-1^+^migratory, but not of Snail2^+^pre-migratory neural crest cells (Fig. 4C). Thus at stage 6, the specification of neural crest progenitor cells has become independent of exposure to both BMP and Wnt signals.

### BMP Activity Maintains the Capacity to Induce Neural Crest Character at Stage 10

In contrast to our results, a recent study argues that Wnt signals, but not BMP signals, induce neural crest cells in explants isolated from the intermediate region of the stage 10 caudal neural plate (C) by monitoring the induction of HNK-1 after 48 hr of culture, corresponding in time to stage ∼20 [11]. HNK-1 is also, however expressed in dorsal spinal cord cells by stage 20 (Fig. S5B), and consistently stage 10 C explants cultured alone for 48 hr, generated Sox1^+^ and HNK-1^+^ cells (Fig. S5C). To re-examine whether BMP and/or Wnt signals induce neural crest character in neural cells at stage 10, we cultured stage 10 C explants for 24 hr, corresponding in time to approximately stage 17, when HNK-1 is expressed in neural crest but not in cells in the dorsal neural tube (Fig. S5A). In addition, we monitored the expression of Sox1 which is expressed in neural cells but not in neural crest cells. When stage 10 C explants were exposed to BMP4 (20 ng/ml) or Wnt3A (2×–4×) in different culture medium with or without N2 supplement, BMP but not Wnt signals inhibited the generation of neural Sox1^+^ cells and induced Snail2^+^ and HNK-1^+^ neural crest cells (Fig. S6). Thus, these results provide evidence that at stage 10, BMP activity still has the capacity of inducing neural crest character in caudal neural cells.

### Wnt Signaling Upregulates *Bmp4* Expression, which Induces Neural Crest in Prospective Forebrain Cells

**Figure 5:**
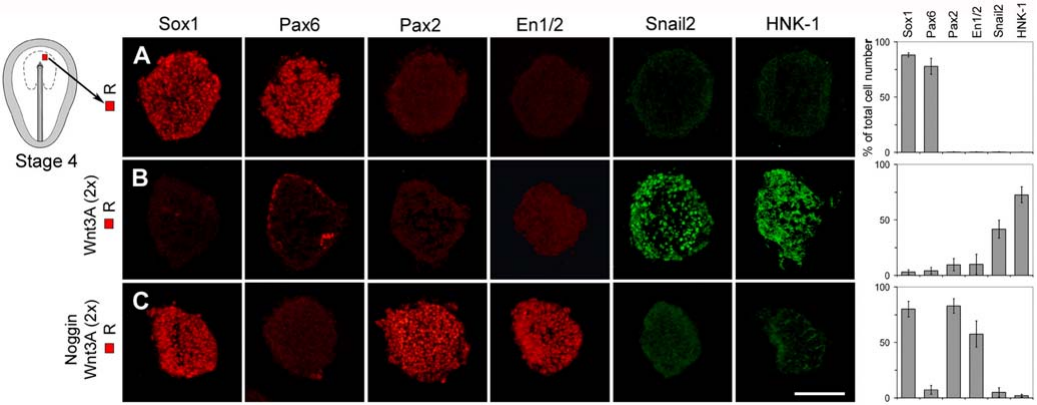
Wnt-regulated BMP Activity Induces Neural Crest Character in Prospective Forebrain Cells. (A–C) Consecutive sections showing expression of molecular markers in explants cultured for 20–22 hr. (A) Stage 4 R explants cultured alone (n = 30) generated Sox1^+^ and Pax6^+^cells, but no Pax2^+^, En1/2^+^, Snail2^+^ or HNK-1^+^ cells. (B) Stage 4 R explants cultured together with Wnt3A (2x) (n = 30) generated Snail2^+^and HNK-1^+^cells, but no Sox1^+^, Pax6^+^, Pax2^+^ or En1/2^+^ cells. (C) Stage 4 R explants cultured together with Wnt3A (2×) and Noggin (n = 20) generated Sox1^+^, Pax2^+^ and En1/2^+^cells, but no Pax6^+^, Snail2^+^ or HNK-1^+^ cells. Data are represented as mean±SEM. Scale bar, 100 µm (A–C).

**Figure 6:**
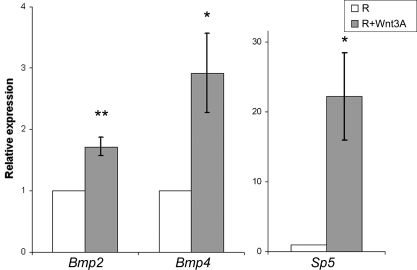
Wnt Signaling Up-regulates *Bmp2* and *Bmp4* Expression in Prospective Forebrain Cells. Relative *Bmp2*, *Bmp4* and *Sp5* mRNA levels measured by quantitative real-time PCR in stage 4 R explants cultured in the presence of Wnt3A (2x) compared to explants cultured alone. Bars represent mean±SEM of 5 independent experiments.

Consistent with the idea that BMP signals induce neural crest character only in cells that have been caudalized by Wnt, forebrain explants exposed to BMP signals generate cells of olfactory and lens placodal character but no cells of neural crest character ([5] and data not shown). These results indicate that sequential or simultaneous exposure of stage 4 prospective forebrain cells to Wnt and BMP signals would generate neural crest cells. To test this possibility we first cultured stage 4 rostral (R) explants, fated to generate cells of forebrain character, alone or in the presence of Wnt3A (2×–4×). Stage 4 R explants cultured alone generated Sox1^+^ and Pax6^+^ neural cells of forebrain character, but no Snail2^+^, HNK-1^+^ or Ker^+^ cells (Fig. 5A), whereas Wnt3A blocked the generation of neural cells, and surprisingly induced Snail2^+^ and HNK-1^+^ neural crest cells (Fig. 5B). No mesodermal cells were detected in explants exposed to Wnt3A (Fig. S3D). At early blastula stages, Wnt signals inhibit neural and induce epidermal fate by promoting BMP signaling [28], [29], raising the possibility that Wnt signals induce neural crest character in prospective forebrain cells by up-regulating BMP activity. To test this possibility, we cultured stage 4 R explants alone or in the presence of Wnt3A (2x) for 10 hr, and monitored *Bmp2* and *Bmp4* mRNA levels by quantitative real-time PCR. *Bmp2 and Bmp4* mRNAs were induced 1.7-fold and 3.0-fold, respectively, in stage 4 R explants exposed to Wnt3A (2x) compared to stage 4 R explants cultured alone (Fig. 6). A ∼20 fold induction of *Sp5* mRNA, a Wnt target gene [30], confirmed the activation of the Wnt pathway in this assay (Fig. 6). Thus, induction of neural crest cells in prospective forebrain cells by Wnt signals correlates with an increase in *Bmp2* and *Bmp4* expression.

To examine whether BMP activity is required for the induction of neural crest cells by Wnt3A, we cultured stage 4 R explants in the presence of Wnt3A (2×) and Noggin. Under these conditions Noggin blocked the generation of Snail2^+^ and HNK-1^+^ neural crest cells and Sox1^+^ neural cells were generated (Fig. 5C). In addition, expression of Pax6 was blocked and Pax2 and En1/2 expression was induced, characteristic of midbrain-hindbrain cells (Fig. 5C). Thus, Wnt activity caudalizes prospective forebrain cells and promotes BMP signaling, which in turn induces neural crest cells. Taken together, these results suggest that BMP activity induces border region cells, which in the Wnt induced caudal region of the embryo are of neural crest character.

## Discussion

In this study we have addressed how the induction and early development of the central and peripheral nervous systems are coordinated. Previous results have provided evidence that graded Wnt signals impose rostrocaudal character on neural plate cells at the late gastrula stage [14], [15]. We now provide evidence that at the late gastrula stage, Wnt signals impose caudal character on neural plate border cells and that under these conditions, BMP signals induce neural crest cells instead of rostral placodal cells. Thus, the status of Wnt signaling in ectodermal cells at the late gastrula stage regulates the rostrocaudal patterning of both neural and neural plate border cells, providing a coordinated spatial and temporal control of the early development of the central and peripheral nervous systems.

**Figure 7:**
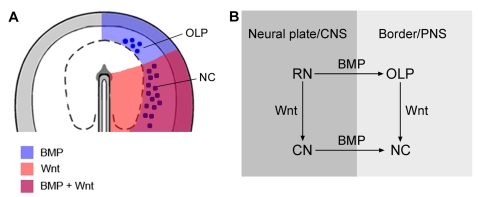
Early Development of the Central and Peripheral Nervous Systems is Coordinated by Wnt and BMP Signals. (A) Schematic representation of a stage 4 embryo showing the distribution of Wnt and BMP activities in the neural plate and neural plate border. (B) Schematic diagram showing proposed roles of Wnt and BMP signals between stage 4 and 6 in chick. Wnt signals impose caudal character in both neural plate and neural plate border cells. At rostral neural (RN) levels in absence of Wnt signals, BMP signals induce olfactory/lens placodal (OLP) cells, and at caudal neural (CN) levels in the context of Wnt signaling, BMP signals induce neural crest (NC) cells.

Neural crest cells are generated along the entire rostrocaudal neuraxis except at rostral forebrain levels, where border cells generate olfactory/lens placodal cells [1]–[3]. Already at the late gastrula stage, cells in the rostral neural plate border are specified as olfactory/lens placodal cells and cells in the caudal border are specified as neural crest cells [4], [5]. At gastrula stages, *Bmp2* and *Bmp4* are expressed in the ectoderm surrounding the entire neural plate [6], domains where phosphorylated Smad-1 is also detected, indicative of activated BMP signaling [7]. In agreement with this pattern of expression, recent results have provided evidence that at the late gastrula stage, BMP signals induce border cells of olfactory and lens placodal character at prospective rostral forebrain levels [5] which do not generate neural crest cells. We now provide evidence that at the late gastrula stage, BMP activity is required and sufficient to induce border cells of neural crest character at caudal levels of the neuraxis. Collectively, our study and previous results [5] provide evidence that BMP signals induce border derivatives of both rostral and caudal character at the appropriate rostrocaudal levels of the neuraxis at stage 4, the late gastrula stage. At stage 5, both pre-migratory neural crest [31] and olfactory/lens placodal cells (Fig. S7A and S7B) still require ongoing exposure to BMP signals, whereas only a few hours later by stage 6, the generation of pre-migratory neural crest and olfactory placodes has become independent of further BMP activity (Fig. S7C and S7D; see also [27]). In summary, these results provide evidence that BMP signals regulate early spatial and temporal development of the peripheral nervous system (Fig. 7).

Our data provide evidence that also at stage 10, after the initial induction of neural crest cells, BMP but not Wnt signals can induce neural crest fate in caudal intermediate neural cells, which is consistent with previous results [10], and in agreement with the finding that caudal neural cells have been exposed and responded to Wnt activity already at stage 4 [15], [32]. A more recent study has argued, however, that Wnt, but not BMP signals induce neural crest character in stage 10 intermediate neural cells [11], although a follow-up study indicated that BMP signals induce neural crest cells more robustly than Wnt signals [12]. A possible explanation for these discrepancies is that Garcia-Castro et al and Taneyhill et al [11], [12] used markers which are expressed both in neural crest cells and also at some stage in neural progenitor cells in the caudal dorsal neural tube [10], [12], [33], but did not monitor loss of neural character, and thus not the shift between neural and neural crest fate. In contrast, using Sox1 as a definitive neural marker, we monitored the fate shift between neural and neural crest cells. Thus, using markers detecting neural crest progenitors, pre-migratory and migratory neural crest cells, as well as marker of neural and epidermal cells, our results provide clear evidence that both at the late gastrula stage, when neural crest cells are initially induced, and by stage 10 after induction of neural crest cells, BMP but not Wnt signals induce neural crest character in caudal neural cells.

Members of the *Wnt* family are expressed in the ectoderm and in the mesoderm in the caudal region of the gastrula stage embryo, and Wnt inhibitors are expressed in more rostral regions of the embryo [13]. Consistent with these patterns of expression, Wnt signals induce caudal character in prospective neural cells at the late gastrula stage [15]. At this stage, BMP signaling is ongoing in both rostral and caudal border cells [6], [7], and our results provide evidence that exposure of rostral border cells to Wnt signals induces the generation of neural crest cells at the expense of olfactory and lens placodal cells. These results are supported by recent findings in *Xenopus* which indicate that the exclusion of neural crest cells from the rostral border is dependent on Dickkopf1, an inhibitor of Wnt signals [34]. In addition, our results provide evidence that attenuation of Wnt signaling in caudal border cells blocks the generation of neural crest cells and promotes the generation of olfactory and lens placodal cells normally generated from rostral border cells. Thus, the role of Wnt signals in the specification of neural crest cells is to impose caudal character on cells in the neural plate border region. In summary, our results provide evidence that at the late gastrula stage the status of Wnt signaling in the border region determines whether BMP activity induces border cells of neural crest or olfactory/lens placodal character (Fig. 7).

The present and previous results [15], [26] also provide evidence that both neural and neural plate border cells acquire caudal character in response to Wnt signals at the late gastrula stage, and that both classes of cells become independent of exposure to further Wnt signals by the head fold stage, stage 6 in chick. These results suggest a temporally coordinated requirement for Wnt signals in the specification of caudal neural plate and caudal border cells. A study in *Xenopus* has reported that the requirement for Wnt signaling in the induction of neural crest cells can be uncoupled from the caudalization of neural plate cells [35]. In this study, inhibition of the Wnt pathway was performed in whole embryos from the two cell stage, which resulted in a reduction of neural crest marker expression, but not of caudal neural markers [35]. Previous studies have provided evidence that the generation of caudal neural cells requires Wnt signaling [14], [15], [36], implying that Wnt signaling in the caudal neural plate was only partially inhibited under the conditions used by Wu et al [35]. Thus, under these conditions Wnt signaling may be more reduced in prospective neural crest cells than in caudal neural cells in these embryos or the generation of neural and neural crest cells requires different levels of Wnt activity. In summary, our results provide evidence that Wnt signals regulate the rostrocaudal patterning of both neural and neural plate border cells, and that Wnt in combination with BMP signals provide a coordinated spatial and temporal control of the early development of the central and peripheral nervous systems (Fig. 7).

## Materials and Methods

### Isolation and Culture of Tissue Explants

Fertilized white leghorn chicken eggs were obtained from Agrisera AB, Umeå, Sweden. Chick embryos were staged according to the protocols of Hamburger and Hamilton [37]. Ectodermal explants were isolated using a tungsten needle. Explants of the prospective neural crest were isolated from stage 4 and 6 chick embryos, and explants of the prospective olfactory/lens placodal, midbrain-hindbrain and forebrain regions were isolated from stage 4 embryos. All explants were cultured in vitro in collagen (Vitrogen) in serum-free conditions. Unless stated, culture media consisted of OPTI-MEM (Gibco) containing N2 supplement (Invitrogen) and fibronectin (Sigma). Wnt3A was used at an estimated concentration of 150 to 600 ng/ml (1×–4×), mFrz8CRD media were used at 50 µl/ml culture medium and Noggin at an estimated concentration of 25 ng/ml. Explants cultured in the presence of control conditioned media generated the same combination of cells as explants cultured alone (Fig. S8). Human BMP4 (R&D Systems) was used at 20–35 ng/ml. The use of chick embryos in this study was approved by the ethical committee at Umeå University.

### In Situ Hybridization and Immunohistochemistry

For in situ RNA hybridization and immunohistochemistry, embryos and explants were fixed as described [23] and serially sectioned at 8–10 µm. In situ RNA hybridization using a chick digoxigenin-labeled *Raldh3* [38] probe was performed essentially as described [39]. For immunohistochemistry the anti-Sox1, the Wide Spectrum Screening anti-Cytokeratin (DakoCytomation), anti-Pax2 (Biosite) rabbit antibodies, the anti-δ-crystallin sheep antibody [40], the monoclonal anti-Slug [10], anti-HuCD (Molecular Probes), anti-HNK-1 [33], anti-Pax6 [41], anti-Pax7 [42] and anti-En1/2 4G11 [41] antibodies were used. Nuclei were stained using DAPI (Sigma).

### Quantitative Real-Time PCR Analysis

Total RNA was derived from cultured forebrain explants (n = 16). Primer sequences were the following: Bmp2: 5′–CGCAGCTTCCACCACGA-3′, 5′-CCCACTTGTTTCTGGCAGTTCT-3′; Bmp4: 5′-GGGCCAACACCGTGAGG-3′, 5′–CAGGTGCTCTTCATGGTGGAA-3′; Sp5: 5′-TGTAAAGCGACCCGCGA-3′, 5′-AACGTATTTATTTTCACGCTGCAA-3′; Gapdh: 5′-CTGTTGTTGACCTGACCTGCC-3′, 5′-TCATACTTGGCTGGTTTCTCCAG-3′; Histone H4: 5′-TCACCTACACCGAGCACGC-3′, 5′-CCGTGACCGTCTTCCTCTTG-3′; S17: 5′-CTACGTGCCCGAGGTCTCTG-3′, 5′-GGGTCCACTTCAATGATCTCCT-3′.

### Statistical Analysis

Consecutive sections from the same explants were stained in multiple ways. The percentage of antigen-expressing cells was quantified by counting the number of stained cells in 2–4 sections per explants (n = 6–9). The total number of cells per section was determined by counting the number of nuclei using DAPI staining. For quantification of Sox1, Snail2, HNK-1, Ker, Pax2 and En1/2 expression, the graphs represent mean number of cells positively stained as percentage of total cell number. For quantification of HuCD, δ-crystallin and *Raldh3* expression, the graphs represent mean number of cells positively stained as percentage of total number of Ker^+^ cells, quantified in adjacent sections. Error bars represent mean±SEM. P-values in figure 6 were obtained using one-sample T-test (^*^p<0.05; ^**^p<0.01).

More detailed information about materials and methods can be found in Text S1 available online.

## Supporting Information

Text S1 Supplementary materials and methods
(0.05 MB DOC)

Figure S1 Explants Generate Neural Crest Cells in Absence of Mesodermal Cells. (A–D) Consecutive sections showing expression of molecular markers in explants cultured for 20–22 hr. (A) Stage 4 CB explants cultured alone (n = 9) generated HNK-1^+^ cells, a few Ker^+^ cells, but no Sox1^+^ cells, or Chordin^+^, Brachyury^+^, Tbx6L^+^ or Raldh2^+^ mesodermal cells. (B) Stage 4 RB explants cultured in the presence of Wnt3A (1x) (n = 8) generated HNK-1^+^ cells, a few Ker^+^ cells, but no Sox1^+^ cells, or Chordin^+^, Brachyury^+^, Tbx6L^+^ or Raldh2^+^ mesodermal cells. (C) Stage 4 C explants cultured in the presence of BMP4 (20 ng/ml) (n = 9) generated HNK-1^+^ cells, a few Ker^+^ cells, but no Sox1^+^ cells, or Chordin+, Brachyury^+^, Tbx6L^+^ or Raldh2^+^ mesodermal cells. (D) Stage 4 R explants cultured in the presence of Wnt3A (2×) (n = 8) generated HNK-1^+^ cells, a few Ker^+^ cells, but no Sox1^+^ cells, or Chordin^+^, Brachyury^+^, Tbx6L^+^ or Raldh2^+^ mesodermal cells. Scale bar, 100 µm (A–D). (E) Transversal sections of a stage 10 chick embryo at the spinal cord level. Sox1 is expressed in neural cells. HNK-1 is expressed in migratory neural crest cells. Ker is expressed in epidermal cells. Chordin and Brachyury are expressed in the notochord. Raldh2 and Tbx6L are expressed in the paraxial mesoderm.
(3.60 MB TIF)

Figure S2 In the Absence of BMP Activity Prospective Neural Crest Cells Acquire a Midbrain-hindbrain Character. (A) Photographs of wholemount stage 4 CB explants in collagen cultured alone for 22 hr or 30 hr (n = 20). After 30 hr migratory cells are clearly visible. Scale bar, 100 µm. (B,C) Consecutive sections showing expression of Pax2 and En1/2 in explants cultured for 22 hr. (B) Stage 4 CB explants cultured alone (n = 20) generated no Pax2^+^ or En1/2^+^ cells. (C) Stage 4 CB explants cultured in the presence of Noggin (n = 20) generated Pax2^+^ and En1/2^+^ cells, characteristic of the midbrain-hindbrain. Scale bar, 100 µm (B,C).
(0.75 MB TIF)

Figure S3 The Levels of Cell Proliferation and Apoptosis are not Affected by BMP or Wnt Activity in vitro. (A–D) No significant differences in cell proliferation or apoptosis were detected in explants cultured alone compared to explants exposed to mFrz8CRD, Noggin, BMP4 or Wnt3A for 10 hr. (A) Stage 4 CB explants (n = 9) cultured alone or together with Noggin generated ∼11% cells expressing cleaved Caspase 3 and ∼3% cells expressing Mpm2. (B) Stage 4 CB explants (n = 15) cultured alone or together with mFrz8CRD generated ∼13% cells expressing cleaved Caspase 3 and ∼2% cells expressing Mpm2. (C) Stage 4 RB explants (n = 8) cultured alone or together with Wnt3A (1×) generated ∼7% cells expressing cleaved Caspase 3 and ∼3% cells expressing Mpm2. (D) Stage 4 C explants (n = 9) cultured alone or together with BMP4 (20 ng/ml) generated ∼13% cells expressing cleaved Caspase 3 and ∼2% cells expressing Mpm2. Data are represented as mean±SEM. Scale bar, 100 µm (A–D).
(1.40 MB TIF)

Figure S4 In the Presence of Wnt3A β-catenin Nuclear Staining is Increased in Prospective Midbrain-hindbrain Cells. (A,B) Consecutive sections showing expression of β-catenin in explants cultured for 6 hr. (A) Stage 4 C explants cultured alone (n = 9) generated no or only a few cells with positive nuclear β-catenin staining. (B) Stage 4 C explants cultured together with Wnt3A (4×) (n = 9) generated an increased number of cells with positive nuclear β-catenin staining (white arrowheads). Scale bar, 100 µm (A,B).
(0.63 MB TIF)

Figure S5 At Stage 20 HNK-1 is Expressed in Spinal Cord Neural Cells. (A) Transversal section of a stage 17 chick embryo at the spinal cord level. HNK-1 is expressed in neural crest cells but not in Sox1^+^ neural cells. (B) Transversal section of a stage 20 chick embryo at the spinal cord level. HNK-1 is expressed in Sox1^+^ neural cells. (C) Stage 10 C explants cultured alone (n = 30) for 48 hr generated Sox1^+^ and HNK-1^+^ cells in the same region. Scale bar, 100 µm.
(0.70 MB TIF)

Figure S6 BMP4 but not Wnt3A Induces Neural Crest in Spinal Cord Cells under Different Culture Conditions. (A–I) Consecutive sections showing expression of molecular markers in explants cultured for 24 hr. (A,B) Stage 10 C explants cultured alone (n = 20) or together with Wnt3A (4×) (n = 20) in OPTI-MEM with N2 supplement generated Sox1^+^ cells, but no Snail2^+^ or HNK-1^+^ cells. (C) Stage 10 C explants cultured together with BMP4 (20 ng/ml) (n = 20) in OPTI-MEM with N2 supplement generated Snail2^+^ and HNK-1^+^ cells, but no Sox1^+^ cells. (D,E) Stage 10 C explants cultured alone (n = 9) or together with Wnt3A (4×) (n = 9) in OPTI-MEM lacking N2 supplement generated Sox1^+^ cells, but no Snail2^+^ or HNK-1^+^ cells. (F) Stage 10 C explants cultured together with BMP4 (20 ng/ml) (n = 9) in OPTI-MEM lacking N2 supplement generated Snail2^+^ and HNK-1^+^ cells, but no Sox1^+^ cells. (G,H) Stage 10 C explants cultured alone (n = 20) or together with Wnt3A (4×) (n = 20) in F12 with N2 supplement generated Sox1+ cells, but no Snail2^+^ or HNK-1^+^ cells. (I) Stage 10 C explants cultured together with BMP4 (20 ng/ml) (n = 20) in F12 with N2 supplement generated Snail2^+^ and HNK-1^+^ cells, but no or a few Sox1+ cells. Scale bar, 100 µm (A–I).
(1.41 MB TIF)

Figure S7 Rostral Border Cells Become Independent of BMP Signals at the Head Fold Stage. (A–D) Consecutive sections showing expression of molecular markers in explants cultured for 43–45 hr. (A) Stage 5 RB explants cultured alone (n = 10) generated a distinct region of Raldh3^+^, HuCD^+^ and Ker^+^ cells; characteristic of olfactory placodal cells and a separate region of cells expressed δ-crystallin and Ker; characteristic of lens cells, but no L5^+^ neural cells were detected. (B) Stage 5 RB explants cultured in the presence of Noggin (n = 10) generated L5^+^ and HuCD^+^ neural cells, but no Raldh3^+^, δ-crystallin^+^ or Ker^+^ cells were detected. (C) Stage 6 RB explants (n = 12) generated a distinct region of Raldh3^+^, HuCD^+^ and Ker^+^ cells and a separate region of cells expressed δ-crystallin and Ker, but no L5^+^ neural cells were detected. (D) Stage 6 RB explants cultured in the presence of Noggin (n = 12) generated Raldh3^+^, HuCD^+^ and Ker^+^ cells; characteristic of olfactory placodal cells, but no δ-crystallin^+^ or L5^+^ cells. Scale bar, 100 µm (A–D).
(1.78 MB TIF)

Figure S8 Control Conditioned Medium do not Affect the Character of Cells Generated in Explants. (A–G) Consecutive sections showing expression of molecular markers in explants cultured for 20–22 hr. (A–C) Stage 4 CB explants cultured alone (n = 30) or in the presence of control CHO (n = 9) or HEK-293-LacZ conditioned media (n = 9) generated Snail2^+^ cells, HNK-1^+^ cells, and a few Sox1^+^ cells and Ker^+^ cells. (D,E) Stage 4 RB explants cultured alone (n = 20) or in the presence of control L-cell conditioned media (n = 20) generated Ker^+^ cells, but no Sox1^+^, Snail2^+^ or HNK-1^+^ cells. (F,G) Stage 4 R explants cultured alone (n = 20) or in the presence of control L-cell conditioned media (n = 20) generated Sox1^+^ cells, but no Snail2^+^, HNK-1^+^ or Ker^+^ cells. Scale bar, 100 µm (A–G).
(2.73 MB TIF)
