# Supplementary material for: Early Development of the Central and Peripheral Nervous Systems Is Coordinated by Wnt and BMP Signals
Source: PLoS One. 2008 Feb 20;3(2):e1625. doi: 10.1371/journal.pone.0001625 (PMC2229838; doi:10.1371/journal.pone.0001625)
Supplement: Supplementary file 1 [file pone.0001625.s001.doc]

Supporting Materials and Methods

Preparation of Factors. Soluble Wnt3A and control conditioned media (CM) were obtained from stably transfected or untransfected mouse L cells, respectively [1]. Soluble mouse frizzled receptor 8 (mFrz8CRD) and control CM were obtained from HEK-293 cells transfected with *mFrz8CRD* or *LacZ* reporter constructs [2] using Gene-PORTER 2 (GTS_{INC}, San Diego, California). Soluble Noggin and control CM were obtained from stably transfected, or untransfected, CHO cells, respectively [3]. Cells were transferred to serum-free OPTI-MEM (Gibco-BRL; mFrz8CRD and Noggin) or DMEM (Gibco-BRL; Wnt3A), and the CM was concentrated with Centriprep 10,000-MWCO filters (Amicon/Millipore, Bedford, Massachusetts).

In Situ Hybridization and Immunohistochemistry. Chick digoxigenin-labeled *Brachyury* [4], *Chordin* [5], *Raldh2* [6] and *Tbx6L* [7] probes were used. The monoclonal anti-L5 [8] rat antibody and anti-PY489-B-catenin [9] were used. Cell proliferation was monitored using a monoclonal anti-Mpm2 antibody (Upstate Inc) which detects cells in M-phase of the cell cycle. Apoptosis was monitored using a rabbit antibody detecting cleaved Caspase 3 (Asp175) (Cell Signaling Technology Inc).

Statistical Analysis. For quantification of Mpm2 and Caspase 3 expression, the graphs represent mean number of cells positively stained as percentage of total cell number per explant (n=7-9). Error bars represent mean ± SEM. P-values in Figure S3 were obtained using two-tailed Student t-test.

Quantitative Real-time PCR Analysis. Total RNA was derived from cultured forebrain explants (n=16) using a NucleoSpin RNA II kit (740955, Macherey-Nagel) and PCR purification kit (PF1001Viogene). cDNA was prepared using Superscript II Reverse Transcriptase (Invitrogen). Real-time PCR analysis was performed using the ABI PRISM 7000 Sequence Detection System and SYBR Green PCR Master Mix (ABI). Oligonucleotide

primers were designed using Primer Express software (Applied Biosystems). The CT value for each gene was the average of a triplicate, and expression levels were normalized using the average of CT values for Gapdh, S17 and Histone H4.

References

1. Shibamoto S, Higano K, Takada R, Ito F, Takeichi M, et al. (1998) Cytoskeletal reorganization by soluble Wnt-3a protein signalling. Genes Cells 3: 659-670.
2. Hsieh JC, Rattner A, Smallwood PM, Nathans J (1999) Biochemical characterization of Wnt-frizzled interactions using a soluble, biologically active vertebrate Wnt protein. Proc Natl Acad Sci U S A 96: 3546-3551.
3. Lamb TM, Knecht AK, Smith WC, Stachel SE, Economides AN, et al. (1993) Neural induction by the secreted polypeptide noggin. Science 262: 713-718.
4. Smith JC, Price BM, Green JB, Weigel D, Herrmann BG (1991) Expression of a Xenopus homolog of Brachyury (T) is an immediate-early response to mesoderm induction. Cell 67: 79-87.
5. Francis-West PH, Parish J, Lee K, Archer CW (1999) BMP/GDF-signalling interactions during synovial joint development. Cell Tissue Res 296: 111-119.
6. Swindell EC, Eichele G (1999) Retinoid metabolizing enzymes in development. Biofactors 10: 85-89.
7. Knezevic V, De Santo R, Mackem S (1997) Two novel chick T-box genes related to mouse Brachyury are expressed in different, non-overlapping mesodermal domains during gastrulation. Development 124: 411-419.
8. Roberts C, Platt N, Streit A, Schachner M, Stern CD (1991) The L5 epitope: an early marker for neural induction in the chick embryo and its involvement in inductive interactions. Development 112: 959-970.
9. Rhee J, Buchan T, Zukerberg L, Lilien J, Balsamo J (2007) Cables links Robo-bound Abl kinase to N-cadherin-bound beta-catenin to mediate Slit-induced modulation of adhesion and transcription. Nat Cell Biol 9: 883-892.
